# mycoCLAP, the database for characterized lignocellulose-active proteins of fungal origin: resource and text mining curation support

**DOI:** 10.1093/database/bav008

**Published:** 2015-03-08

**Authors:** Kimchi Strasser, Erin McDonnell, Carol Nyaga, Min Wu, Sherry Wu, Hayda Almeida, Marie-Jean Meurs, Leila Kosseim, Justin Powlowski, Greg Butler, Adrian Tsang

**Affiliations:** ^1^Centre for Structural and Functional Genomics, ^2^Department of Computer Science and Software Engineering, ^3^Department of Chemistry and Biochemistry, and ^4^Department of Biology Concordia University, Montréal, QC, USA

## Abstract

Enzymes active on components of lignocellulosic biomass are used for industrial applications ranging from food processing to biofuels production. These include a diverse array of glycoside hydrolases, carbohydrate esterases, polysaccharide lyases and oxidoreductases. Fungi are prolific producers of these enzymes, spurring fungal genome sequencing efforts to identify and catalogue the genes that encode them. To facilitate the functional annotation of these genes, biochemical data on over 800 fungal lignocellulose-degrading enzymes have been collected from the literature and organized into the searchable database, mycoCLAP (http://mycoclap.fungalgenomics.ca). First implemented in 2011, and updated as described here, mycoCLAP is capable of ranking search results according to closest biochemically characterized homologues: this improves the quality of the annotation, and significantly decreases the time required to annotate novel sequences. The database is freely available to the scientific community, as are the open source applications based on natural language processing developed to support the manual curation of mycoCLAP.

**Database URL:** http://mycoclap.fungalgenomics.ca

## Introduction

Plant biomass is the most abundant renewable material on the planet and is an enormous store of sugars. Polysaccharides constitute the bulk of plant biomass. In secondary plant cell wall, the aromatic polymer lignin forms a network of linkages with the polysaccharides to give plant cell wall its rigidity. Plant polysaccharides can be broadly divided into two groups: (i) plant cell wall polysaccharides which are composed of cellulose, hemicelluloses (primarily xylan and mannan) and pectin; and (ii) storage polysaccharides which comprise starch and inulin. Fungi are the major decomposers of plant biomass in the biosphere and they use plant biomass as their predominant carbon source. Fungi cannot import polysaccharides into the cell, but instead secrete a diverse set of enzymes to breakdown the polysaccharides extracellularly into monomeric or oligomeric sugars. The enzymes that microorganisms, and fungi in particular, use to hydrolyze polysaccharides have been used extensively in industrial applications including feed, food, beverages, detergent, textile and pulp and paper. In response to environmental and energy security concerns, there is a recent surge in interest in the production of alternative fuels and chemicals from plant biomass. This has led to an intensification of research into fungal decomposition of plant biomass. In search of diverse fungal enzymes, over 400 fungal genomes have been or are being sequenced (http://genomeonline.org). Many of the sequenced fungal genomes have been predicted to possess 200–600 genes encoding biomass-degrading enzymes (1–5). To date, only a small fraction of these predicted enzymes have been characterized biochemically.

The structure and organization of plant biomass are complex. As a result, a diverse set of enzymes are required to deconstruct plant biomass into monomeric sugars. Based on their modes of action, these enzymes have been grouped into glycoside hydrolases (GH), polysaccharide lyases (PL), carbohydrate esterases (CE) and auxiliary activities (AA). The Carbohydrate-Active enZymes database (CAZy; http://www.cazy.org) classifies the biomass-degrading enzymes into families and sub-families based on the sequence similarity of their catalytic modules (6). As of 2014, the CAZy database lists 113 GH families, 19 PL families and 16 CE families. However, the CAZy database does not provide publicly available bioinformatic tools to predict function or classification of putative CAZymes. The bioinformatic tools to classify CAZymes can be found in resources such as dbCAN (http://csbl.bmb.uga.edu/dbCAN/). Each of these protein families often includes multiple enzyme activities. The current CAZy classification does not distinguish distinct enzyme activities within a family. With the large quantities of sequence data being generated by fungal genome sequencing efforts, high quality functional annotation of biomass-degrading enzymes has become increasingly important. To facilitate the functional annotation of plant biomass-degrading enzymes, we have started to manually curate fungal genes encoding biochemically characterized GH, PL and CE enzymes. This dataset has been used to create a database called mycoCLAP (http://mycoclap.fungalgenomics.ca), a searchable resource for Characterized Lignocellulose-Active Proteins of fungal origin (7). We have since expanded the curation of this dataset of 453 entries (7) to 804 entries in the latest version. We have also developed two open source applications based on NLP to support the manual curation of mycoCLAP. They are publicly released under the MIT license (https://github.com/TsangLab/).

Here, we describe the updated curation since the last publication (7) and the current features of mycoCLAP.

## Data collection and content

### Methods

We use a semi-automated process to identify articles relevant to mycoCLAP, including weekly PubMed (http://www.ncbi.nlm.nih.gov/pubmed) searches using key words such as ‘glycoside hydrolase AND fungus’. Literature is also collected from Google Scholar and the online resources CAZy (6) and BRENDA (8) that maintain extensive information on biomass-degrading enzymes. The full articles are then manually reviewed by our in-house curators. To qualify as an entry for mycoCLAP:

1. the gene and/or protein sequence must be deposited in a public repository,
2. the gene product must be assayed for enzyme activity,
3. the biochemical properties of the enzyme must be reported in a peer-reviewed journal,
4. the enzyme must be extracellular.

We encourage authors with publications that meet these requirements to submit the enzyme name and literature information directly to us for curation. A ‘new data submission’ form is available on our website. If an article is approved for mycoCLAP, the experimental information is recorded in a spreadsheet (7). Where available, the information may include the kinetics parameters, substrate specificity and the pH and temperature optima. Nucleotide and/or amino acid sequences are then automatically retrieved from GenBank (9) and UniProt (10) using the accession number provided in the literature and uploaded into mycoCLAP. If no accession number is available, the article is not selected. Each entry in mycoCLAP represents a unique sequence meaning that if two enzymes from the same source organism differ by even one amino acid, then these will appear as two separate entries in the database. Gene ontology (GO) terms describing the molecular function, biological process and subcellular compartment (11) and Enzyme Commission (EC) numbers (8) are assigned to each enzyme based on experimental data reported in the literature. Every enzyme is then assigned a unique entry identifier using a standardized system specific to mycoCLAP (7).

### Unique entry names

The most common gene naming convention used by authors is a three-letter code representing the encoded protein activity followed by a letter or a number to distinguish each gene from others encoding the same enzyme in a given species. Hence, we assign a three-letter code for each entry by selecting the most commonly used activity codes in the literature. For example, glucoamylase genes have been referred to by the three-letter code glu, gla or gaa. Since gla is the most commonly used three-letter code, it is the one assigned to all glucoamylase entries in mycoCLAP. For genes encoding bi-functional enzymes, the three-letter mycoCLAP code begins with the letter ‘z’ with the following two letters being taken from the two activities of the encoded protein. For example, a bifunctional endoglucanase/xylanase is assigned the code ‘zex’.

The three-letter activity code is followed by a number representing the CAZy family to which the protein belongs. For example, a glucoamylase belonging to GH family 15 is named *gla15*, followed by a letter to distinguish it from other genes encoding the same protein in one species (*gla15A*, *gla15B*, *gla15C* and so on). If the gene name in the article contains a letter, that letter is kept in the entry name but if authors used a number, that number is converted to the corresponding letter. For example, a glucoamylase belonging to GH family 15 that is named *gla1* in the literature is entered as *gla15A* in mycoCLAP. If different glucoamylases from the same CAZy family are named gla1 in the literature, then the letter is assigned in the order of the publication dates of the literature. Therefore, a *gla1* from an article published in 2005 is assigned *gla15A*, while a different *gla1* published in 2010 is assigned *gla15B* (or the next available letter if ‘B’ is taken).

**Figure 1.**
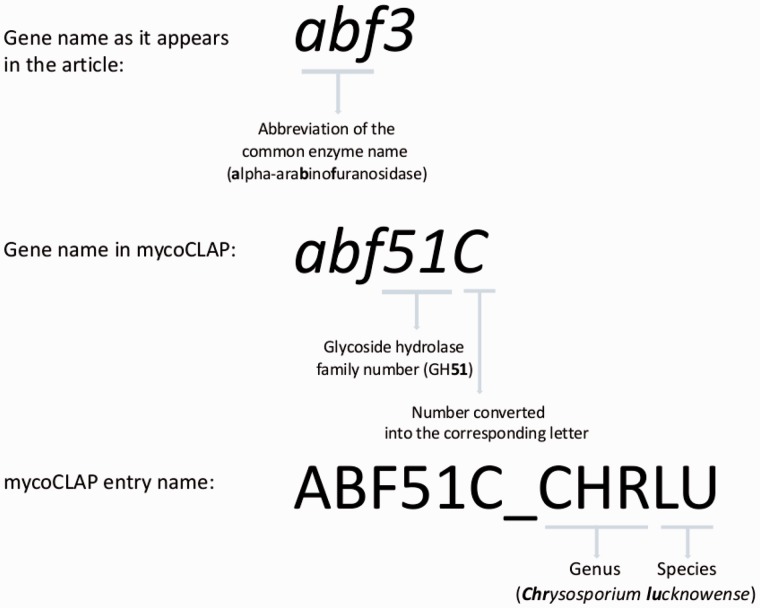
The mycoCLAP entry names.

The assignment of the natural source organism to each entry name is done using the same method as that of UniProt entries, whereby an underscore is added after the gene name followed by a five-letter code. The first three letters represent the genus of the organism and the last two letters represent the species. For the species names, we use the primary name in NCBI taxonomy. If the previously mentioned glucoamylase gene were from *Aspergillus oryzae*, the assigned entry name would be GLA15A_ASPOR. If two species have the same five-letter code, e.g. ASPKA for *A. kawachii* and *A. kassunensis*, another unique letter from one of the species names is used. In the case of *A. kawachii* and *A. kassunensis*, the entries would be ASPKA and ASPKS respectively. In Fig. 1, the GH family 51 alpha-arabinofuranosidase from *Chrysosporium lucknowense* (12) is used as an example to show how entry names for mycoCLAP are constructed.

### Content

The number of entries in mycoCLAP has increased since the database was created in 2011 (7). A total of 804 characterized lignocellulose-active proteins of fungal origin have been collected as of 18 December 2014. These enzymes come from 226 different fungal species, the majority of which are from the phylum Ascomycota. There are 737 GH in the database. Only four of these are classified as bifunctional while the others represent 56 different GH activities and cover 50 of the GH families described in CAZy.

The CEs are far fewer in number with a total of 25. They cover 6 of the 16 CE families described by CAZy and represent 6 different activities. There are currently 9 characterized PL in mycoCLAP. They come from three CAZy PL families and represent three different activities including pectin-, pectate- and rhamnogalacturonan lyase activity.

The collection of enzymes in mycoCLAP has also been expanded to include enzymes with auxiliary activities. Currently, there are 33 enzymes in the database which come from four AA families described in CAZy. A summary of all of the characterized enzymes in mycoCLAP is provided in Table 1 and on our website under Data Summary.

**Table 1:** Family activity summary

Family	Number of characterized enzymes	Activity
Category: auxiliary activities		
AA2	20	Versatile peroxidase(3), peroxidase(2), manganese peroxidase(6), lignin peroxidase(9)
AA3	7	Pyranose 2-oxidase(1), glucose oxidase(4), aryl-alcohol oxidase(2)
AA5	1	Glyoxal oxidase(1)
AA9	5	Polysaccharide monooxygenase(5)
Category: bifunctional glycoside hydrolases		
GH11/CE1	1	Bifunctional xylanase/acetylxylan esterase(1)
GH43	1	Bifunctional alpha-arabinofuranosidase/beta-xylosidase(1)
GH5	1	Bifunctional endoglucanase/xylanase(1)
GH54	1	Bifunctional alpha-arabinofuranosidase/beta-xylosidase(1)
Category: carbohydrate esterases		
CE1	12	Feruloyl esterase(8), acetylxylan esterase(4)
CE12	1	Rhamnogalacturonan acetylesterase(1)
CE4	4	Chitin deacetylase(3), acetylxylan esterase(1)
CE5	5	Cutinase(3), acetylxylan esterase(2)
CE6	1	Acetylxylan esterase(1)
CE8	2	Pectin methylesterase(2)
Category: GH		
GH1	10	Beta-glucosidase(10)
GH10	34	Tomatinase(1), xylanase(33)
GH11	75	Endo-beta-1,4-xylanase(1), xylanase(74)
GH115	1	Xylan alpha-1,2-glucuronidase(1)
GH12	35	Xyloglucanase(5), licheninase(1), endoglucanase(29)
GH128	1	Glucan endo-1,3-beta-d-glucosidase(1)
GH13	27	Oligo-1,6-glucosidase(4), alpha-glucosidase(4), alpha-amylase(19)
GH132	1	Exo-1,3-beta-glucanase(1)
GH15	20	Glucoamylase(20)
GH16	11	Licheninase(2), laminarinase(2), endo-beta-1,3-galactanase(1), mixed-link glucanase(6)
GH17	2	Laminarinase(1), exo-1,3-beta-glucanase(1)
GH18	34	Chitinase(33), endo-*N*-acetyl-beta-d-glucosaminidase(1)
GH2	7	Exo-glucosaminidase(2), beta-mannosidase(3), beta-galactosidase(2)
GH20	12	Hexosaminidase(12)
GH26	5	Beta-mannanase(5)
GH27	16	Alpha-galactosidase(16)
GH28	65	Alpha-l-rhamnosidase(1), rhamnogalacturonan hydrolase(1), exo-polygalacturonase(10), endo-rhamnogalacturonase(4), endo-polygalacturonase(48), xylogalacturonase(1)
GH3	41	Beta-xylosidase(10), beta-glucosidase(27), avenacinase(3), tomatinase(1)
GH30	5	Xylanase(1), endo-1,6-beta-glucanase(4)
GH31	16	Alpha-glucosidase(12), invertase(1), glucoamylase(1), alpha-xylosidases(1), alpha-xylosidase(1)
GH32	23	Invertase(12), exo-inulinase(7), endo-inulinase(4)
GH32/GH43	4	Endo-inulinase(2), exo-inulinase(2)
GH33	1	Exo-alpha-sialidase(1)
GH35	5	Exo-beta-1,4-galactanase(1), beta-galactosidase(4)
GH36	8	Alpha-galactosidase(8)
GH43	17	Alpha-arabinofuranosidase(1), beta-xylosidase(5), exo-beta-1,3-galactanase(2), arabinoxylan arabinofuranohydrolase(2), exo-1,3-beta-galactanase(1), endo-1,5-alpha-arabinanase(6)
GH45	16	Endoglucanase(16)
GH47	6	Alpha-1,2-mannosidase(6)
GH49	4	Isopullulanase(1), dextranase(3)
GH5	78	Beta-mannanase(20), endoglucanase(39), endo-1,6-beta-glucanase(3), endo-1,6-beta-galactanase (galactanase)(2), exo-1,3-beta-glucanase(14)
GH51	11	Alpha-l-arabinofuranosidase(1), alpha-arabinofuranosidase(10)
GH53	7	Arabinogalactanase(7)
GH54	12	Alpha-arabinofuranosidase(11), alpha-l-arabinofuranoside arabinofuranohydrolase(1)
GH55	10	Laminarinase(3), exo-1,3-beta-glucanase(7)
GH6	20	Endoglucanase(2), cellobiohydrolase(18)
GH61	3	Cellulase-enhancing protein(3)
GH62	8	Alpha-arabinofuranosidase(4), arabinoxylan arabinofuranosidase(2), arabinoxylan arabinofuranohydrolase(1), alpha-l-arabinofuranosidase(1)
GH65	2	Trehalase(2)
GH67	6	Xylan alpha-1,2-glucuronidase(1), alpha-glucuronidase(5)
GH7	39	Cellobiohydrolase(25), xylanase(1), mixed-link glucanase(1), endoglucanase(12)
GH71	6	Mutanase(6)
GH74	6	Xyloglucanase(4), oligoxyloglucan cellobiohydrolase(2)
GH75	8	Chitosanase(8)
GH78	6	Alpha-l-rhamnosidase(6)
GH79	1	Beta-glucuronidase(1)
GH81	3	Laminarinase(3)
GH84	1	Endo-beta-*N*-acetylglucosaminidase (*N*-acetylglucosaminidase)(1)
GH85	1	Endo-beta-*N*-acetylglucosaminidase(1)
GH9	1	Endoglucanase(1)
GH93	2	Exo-arabinanase(2)
Category: PL		
PL1	4	Pectin lyase(2), pectate lyase(2)
PL3	3	Pectate lyase(3)
PL4	2	Rhamnogalacturonan lyase(2)

## Web interface

**Figure 2.**
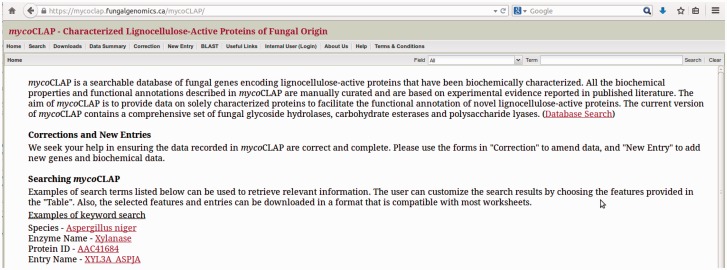
The mycoCLAP homepage.

A web-based system named mycoCLAP (http://mycoclap.fungalgenomics.ca) has been implemented for users to access information on characterized lignocellulose-active enzymes in a user-friendly manner. Three major functions are provided including searching for characterized enzymes, data/sequence retrieval, and BLAST (13) to compare a query sequence to the mycoCLAP sequence collection. Users can find ‘Help’ pages describing the information contained in mycoCLAP on the website, including tips on how to search the database. A screenshot of mycoCLAP homepage is presented in Fig. 2.

### mycoCLAP search and tabular output

**Figure 3.**
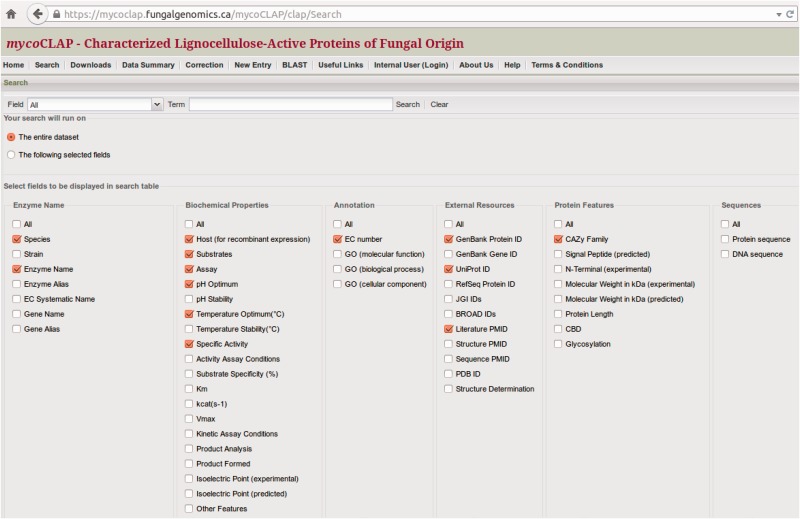
Configuration of the data table. Selected fields will be displayed in the search-results table.

At the top of the mycoCLAP homepage, users may search the database using a gene or protein name, Genbank accession number, UniProt ID, CAZy family number, species, EC number, PubMed ID, keywords or short phrases. The keyword search can include Boolean operators (AND, OR, NOT) so that mycoCLAP can retrieve all enzymes having identifiers or descriptions containing the keywords. For instance, a search for the keyword ‘xylan*’ will retrieve data with descriptions of ‘xylan’, ‘xylanase’, etc., while ‘xylan AND Piromyces’ will retrieve data containing both terms ‘xylan’ and ‘Piromyces’ in their descriptions. Example search terms are shown on the homepage to help users get started. Another built-in search feature that may be helpful in making queries is the ‘search’ tab that allows one to customize the data output table. This tab lists pre-defined options that can be selected or de-selected before launching a search (see Fig. 3). The parameters are automatically reset to the default preferences when a new search is performed.

**Figure 4.**
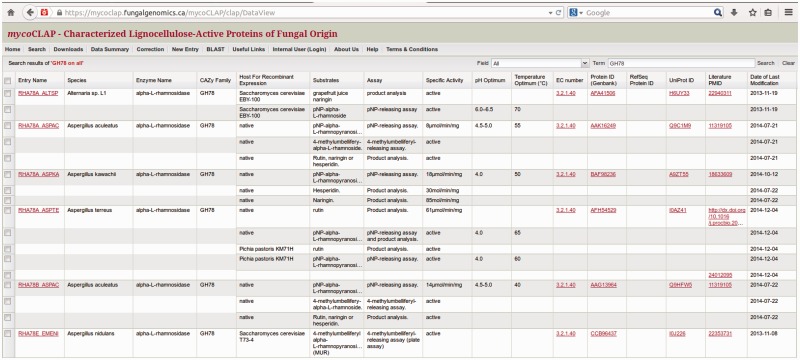
The GH78 enzymes in mycoCLAP.

The search results in mycoCLAP are presented in tabular format. For example, Fig. 4 shows the search results of all GH78 found in the CAZy family field. Each column contains relevant structural and biochemical data extracted from published literature. mycoCLAP also provides hyperlinks to GenBank, UniProt, PubMed, ExPASy Proteomics Server and GO. Likewise, UniProt now also cross-links to corresponding entries in mycoCLAP.

**Figure 5.**
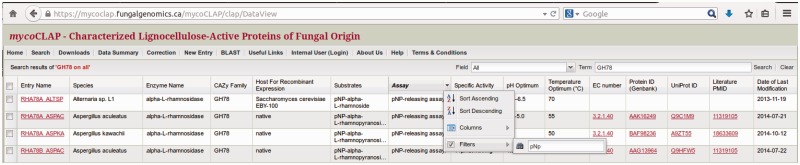
Filtered view in mycoCLAP.

By default, the search-results page lists all matching entries and displays the following fields: mycoCLAP Entry Name, Source Species, Enzyme Name, CAZy Family, Host used for recombinant expression, Substrate(s) and Assay(s) used to measure enzyme activity, Specific Activity in international units, pH and Temperature Optima, EC number, GenBank Protein ID, UniProt ID, literature PubMed ID and Date when the entry was last modified. In the tabular view, users can refine a search by clicking on any column header in the data table, moving the mouse to the empty field next to ‘Filters’ in the drop-down menu, and typing in the search term as shown in Fig. 5.

mycoCLAP provides a full-text search implemented by Lucene indexing search engine (http://lucene.apache.org/). Some fields including the species, GH family, EC number and the expression system are also indexed to improve search results. mycoCLAP supports all Lucene query syntax, including keyword matching and wildcard matching. It also provides quick search on a few specific fields such as species, CAZy family, EC number and Host.

The search function in mycoCLAP is case-insensitive.

### Enzyme page

From the data table the user may select an entry name. This will open the corresponding enzyme page in a new tab. The enzyme page is divided into eight sections:

1. Name and origin. This part includes the assigned mycoCLAP gene name, alternate gene and enzyme names as they appear in the literature, a definition of the enzyme, the source organism from which the enzyme was cloned or isolated, and a brief description of the activity catalyzed by the enzyme.
2. Biochemical properties. This section includes the specific activity, kinetic constants and the substrate specificities (in cases where multiple substrates were evaluated) as reported in the literature.
3. Enzyme annotation. This section contains the EC numbers with a link to the ExPASy proteomics server and GO annotations which link to the GO Consortium website.
4. Literature. This part displays the title of the article(s) related to the entry, the list of authors and their affiliations and the abstract. Each citation also includes a link to PubMed if one is available. The digital object identifier name is alternatively displayed if an article is not in NCBI.
5. Protein features. This section gives the length of the signal peptide as predicted by SignalP 4.0 (14). It also indicates whether the N-terminal sequence of the protein has been experimentally determined. Information on the carbohydrate-binding domain, glycosylation state and CAZy classification can also be found here.
6. Sequences. The nucleic acid sequence and/or the amino acid sequence are displayed here.
7. Cross-references. In this section, links to external resources such as GenBank and UniProt are provided.
8. Entry history. The date when an entry was last modified appears here.

### Data download

Data in mycoCLAP can be downloaded as a text file in tab-delimited format. Users can download an entire dataset or a subset of the data. To download only a subset of the data, one can use the ‘Search’ function to retrieve the dataset of interest. Clicking the ‘Download’ button at the bottom left of the data table will download the data in tab-delimited format. Enzyme sequences can be downloaded in FASTA format from the results table or from the enzyme page by clicking on the ‘FASTA’ button. To retrieve all the data in the database, a ‘Downloads’ page is available on the menu bar at the top of the homepage (see Fig. 2).

### BLAST

On the mycoCLAP homepage, clicking the ‘BLAST’ tab located in the menu bar will take the user to the BLAST page. Here, one can BLAST against all sequences in mycoCLAP to determine whether an enzyme of interest is in the database. BLAST can also be used with an unknown query sequence for annotation based on sequence similarity. The BLAST output contains links to the enzyme pages of the matching mycoCLAP entries.

### Implementation

The data in mycoCLAP are managed by a MySQL relational database system. The web interface is implemented using ExtJS, a client-side JavaScript web application framework developed by Sencha (http://www.sencha.com/). The web application is developed using the SoenEA framework (http://soenea.htmlweb.com/) which is a Java framework that runs on the Apache Tomcat web server.

## NLP tools

Two applications based on NLP have been developed to support the manual curation of mycoCLAP: the mycoMINE, and the mycoSORT systems.

### mycoMINE

mycoMINE (15) is a NLP pipeline that supports the extraction of relevant information from literature related to fungal enzymes. The types of extracted entities have been defined by the curators according to the information needed in mycoCLAP. For instance, mycoMINE extracts names of enzymes, assays, genes, substrates, as well as catalytic properties, and protein properties. For enzyme recognition, the system relies on external resources, BRENDA and SwissProt/UniProtKB, that provide the enzyme knowledge, thus allowing mycoMINE to annotate the extracted enzyme information with additional content as EC numbers, recommended names, scientific names, etc. The enzyme recognition process is rule-based: Gazetteer and mapping lists are automatically extracted from the BRENDA database, in addition to a mapping list of SwissProt identifiers extracted from the SwissProt database. The pipeline makes use of logical rules, gazetteer lists of specific vocabulary and mapping lists to detect key mentions at the entity or the sentence levels. The mycoMINE implementation is based on the GATE framework (16). The mycoMINE source code is publicly available as an open source toolkit (https://github.com/TsangLab/mycoMINE).

While mycoMINE was developed as a rule-based application because there was not enough material to learn from at the beginning of the curation task, mycoSORT has been designed to support the triage with a machine learning approach.

### mycoSORT

The mycoSORT system (17) is a tool created to support the triage task of the mycoCLAP manual curation process. When gathering information about characterized GH enzymes in biomedical databases, curators usually examine an extensive amount of published literature to finally select, on average, only 10% of curatable documents among all their search results. The use of a system to assist curators in the document triage is beneficial to the entire curation process, since this specific task can represent a bottleneck in the workflow (18).

The objective of mycoSORT is to help with the triage of candidate articles for the mycoCLAP database. mycoSORT learns from abstracts that were manually inspected and correctly labeled by biocurators, and outputs a prediction for new abstracts that need to be classified as curatable or non-curatable. mycoSORT uses a set of discriminative features, such as important units of text, to predict the relevance of a particular abstract being chosen to further curation.

A challenging aspect of the automatic triage classification task is the imbalanced class distribution of the dataset. This condition arises due to the fact that the majority of documents retrieved in a database search for relevant keywords will usually be rejected by curators. Usually only a very small portion is considered relevant and carried through the curation process. To deal with the imbalance issue, we applied the data undersampling technique (19) with multiple sampling factors, with the goal of decreasing the number of document instances belonging to the majority class and having a more equal distribution of both curatable and non-curatable documents in our training dataset.

**Figure 6.**
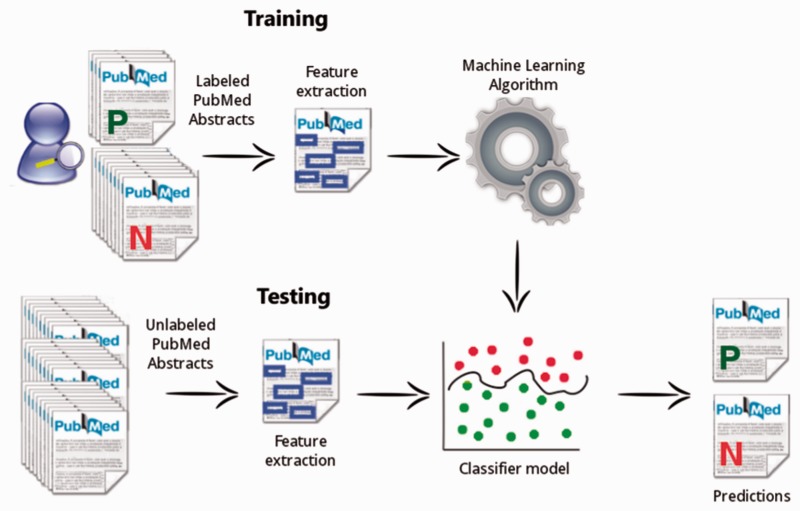
The mycoSORT system workflow.

The mycoSORT workflow is described in Fig. 6. A training dataset, composed by manually labeled PubMed abstracts, is used to extract significant features and to learn the classification model. Once built, the classification model is applied in the testing phase. At this time, the algorithm applies the information learned previously to predict the relevance of a new document instance that is not yet labeled.

When building our classification models, we also experimented with different feature sets and classification algorithms. To extract information from the documents, we applied the text-mining tool mycoMINE (15) to annotate significant bio-entities found in the paper abstract or title. These relevant units of text were used in a combined way to identify a suitable composition that resulted in a more effective classifier for triage. In total, we experimented with 3 machine learning algorithms, 4 feature settings and 9 under-sampling factors, totaling 108 experiments (17).

The mycoSORT system source code has been fully implemented, and is publicly available as an open source toolkit (https://github.com/TsangLab/Annotators). mycoSORT can be applied on the literature triage of lignocellulose-active proteins of fungal origin, as well as on the literature triage of other biomedically related subjects. A variety of text annotation schemas is available, such as the Medical Subject Headings vocabulary, the GO and the Unified Medical Language System thesaurus, and they can be used to identify and extract relevant features for different research contexts.

## Conclusion

The mycoCLAP database is intended to facilitate and improve the annotation of novel enzymes active in the decomposition of plant biomass. It provides a means for comparing novel sequences to a set of sequences whose gene products have been experimentally characterized. Such comparisons provide comparators with known properties and should reduce the number of false positives in homology searches. Along with the open source text mining systems developed for supporting the manual curation, these resources should speed up the process of functional annotation and target identification to guide biochemical analyses.

Future work will focus on increasing the collection of GH, CEs, PL and oxidoreductases in mycoCLAP as well as expanding the database to include other enzymes such as proteases and lipases. We will also create new entries with information on genetically engineered versions of characterized enzymes. Through the ongoing efforts of our biocurators and the collaboration of researchers in the field who are willing to make new submissions to the database, mycoCLAP will continue to be regularly updated, thus providing the fungal research community with the latest and most comprehensive collection of experimentally characterized proteins relevant to biomass degradation.
